# 579. Safety and Immunogenicity of Mpox Vaccination in Adolescents

**DOI:** 10.1093/ofid/ofae631.017

**Published:** 2025-01-29

**Authors:** C Mary Healy, C Buddy Creech, Sharon E Frey, Andrea Lerner, Kay Tomashek, John H Beigel

**Affiliations:** Baylor College of Medicine, Houston, TX; Vanderbilt University Medical Center, Nashville, TN; Saint Louis University, St. Louis, Missouri; DMID/NIAID/NIH, Rockville, Maryland; National Institutes of Health, Rockville, MD; The National Institute of Allergy and Infectious Diseases, National Institutes of Health, Rockville, Maryland

## Abstract

**Background:**

Monkeypox (Mpox) is a global public health threat. In the current Democratic Republic of Congo (DRC) mpox outbreak, children < 15 years of age comprise 70% of cases and 88% of deaths. Modified Vaccinia Ankara-Bavarian Nordic (MVA-BN) vaccine is a live attenuated, non-replicating orthopoxvirus vaccine licensed in the US to prevent smallpox and mpox. MVA-BN is not approved in persons < 18 years of age (though is available in some countries under emergency use authorization).

Geometric Mean Titers of Vaccinia-Specific PRNT by Time Point and Age Group

**Figure:**
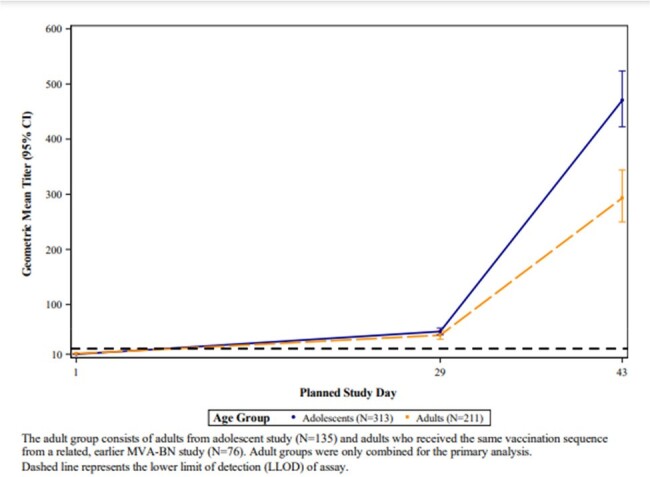

**Methods:**

We conducted a Phase 2, open-label, multisite clinical trial to evaluate the safety and immunogenicity of two 1x10^8^ TCID_50_ doses of MVA-BN vaccine administered subcutaneously 28 days apart in adolescents aged 12-17 years compared to adults aged 18-50 years (NCT05740982). A planned interim analysis evaluated safety through Day 210 (180 days post dose 2) and immunogenicity through Day 43 (14 days post dose 2).

**Results:**

Three hundred fifteen adolescents (161 [51%] aged 12-14 years, 160 [51%] male, 216 [69%] white, 251 [80%] not Hispanic or Latino) were compared to 211 adults **(**94 [45%] male, 145 [69%] white, 149 [71%] not Hispanic or Latino). Solicited systemic and local events, and unsolicited adverse events (AEs), were similar in both groups. Dizziness was more common in adolescents (9 events in 8/315 (3%) versus none in adults) but similar to rates reported after other adolescent vaccines. Overall, MVA-BN was well tolerated. Day 43 antibody responses, based on vaccinia virus (Western Reserve strain) plaque-reduction neutralization titer (PRNT) assay geometric mean titer (GMT), elicited by MVA-BN in adolescents (470.3 [95% CI: 422.3, 523.8]) were non-inferior to the response in adults (293.2 [95% CI: 249.8, 344.2]); GMT ratio: 1.60 [95% CI: 1.32, 1.95]) (Figure).

**Conclusion:**

The interim data demonstrate MVA-BN vaccine is well-tolerated and the peak GMT met prespecified non-inferiority criteria for adolescents as compared to adults. These findings are relevant to adolescents in the US and in areas where Mpox is endemic. Evaluations in younger children are urgently needed to extend protection to those who are most vulnerable.

**Disclosures:**

**C. Mary Healy, MD, FIDSA**, Dexcom Inc: Stocks/Bonds (Public Company)|Hillevax, Inc: Member of Advisory board|Intuitive Surgical Inc: Stocks/Bonds (Public Company)|Quidel Corporation: Stocks/Bonds (Public Company)|Vapotherm: Stocks/Bonds (Public Company) **C. Buddy Creech, MD, MPH**, CommenseBio: Advisor/Consultant|GSK: Advisor/Consultant|Moderna: Advisor/Consultant|Moderna: Grant/Research Support|TDCowen: Advisor/Consultant|UpToDate: Honoraria|Vedanta: Grant/Research Support **Sharon E. Frey, MD**, Bavarian Nordic: Grant/Research Support|Saint Louis University (SLU): Grant/Research Support

